# Ancient DNA from a 2700-year-old goitered gazelle (*Gazella subgutturosa*) supports gazelle hunting in Iron Age Central Asia

**DOI:** 10.1098/rsos.220104

**Published:** 2022-06-15

**Authors:** André Elias Rodrigues Soares, Nikolaus Boroffka, Oskar Schröder, Leonid Sverchkov, Norbert Benecke, Torsten Günther

**Affiliations:** ^1^ Human Evolution Program, Department of Organismal Biology, Uppsala University, Uppsala, Sweden; ^2^ Eurasia Department, German Archaeological Institute, Berlin, Germany; ^3^ Department of Natural Sciences, German Archaeological Institute, Berlin, Germany; ^4^ Department of Terrestrial Zoology, Senckenberg Research Institute and Natural History Museum, Frankfurt am Main, Germany; ^5^ Institute of Fine Arts, Academy of Sciences of the Republic of Uzbekistan, Tashkent, Uzbekistan

**Keywords:** ancient DNA, mitogenome, Iron Age, Central Asia, archaeogenomics

## Abstract

Central Asia has been an important region connecting the different parts of Eurasia throughout history and prehistory, with large states developing in this region during the Iron Age. Archaeogenomics is a powerful addition to the zooarchaeological toolkit for understanding the relation of these societies to animals. Here, we present the genetic identification of a goitered gazelle specimen (*Gazella subgutturosa*) at the site Gazimulla-Tepa, in modern-day Uzbekistan, supporting hunting of the species in the region during the Iron Age. The sample was directly radiocarbon dated to 2724–2439 calBP. A phylogenetic analysis of the mitochondrial genome places the individual into the modern variation of *G. subgutturosa*. Our data do represent both the first ancient DNA and the first nuclear DNA sequences of this species. The lack of genomic resources available for this gazelle and related species prevented us from performing a more in-depth analysis of the nuclear sequences generated. Therefore, we are making our sequence data available to the research community to facilitate other research of this nowadays threatened species which has been subject to human hunting for several millennia across its entire range on the Asian continent.

## 1. Introduction

**Figure 1.**
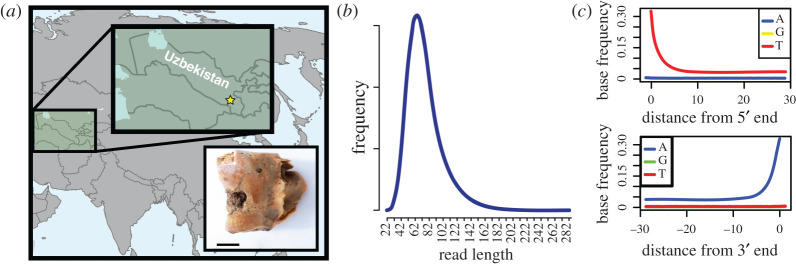
(*a*) Locality in which the bone fragment was found is indicated on the map with a yellow star. The inset contains a photo of the bone fragment (sample AGAZ005, Gazimullah Y:2006 F:3). Black bar indicates 1 cm length. (*b*) DNA fragment length distribution short sequences characteristic of ancient DNA. (*c*) Misincorporation plots indicating DNA damage patterns towards the end of the sequencing reads, also congruent with authentic ancient DNA fragments.

The Surkhandary Region in southeastern Uzbekistan as part of the ancient region of Bactria has been a focus for archaeological research for many years. Belonging to the Achaemenid Empire, the ‘First Persian Empire’, it was later conquered by Alexander the Great. Excavations at the fortress of Kurganzol produced well-stratified samples of many faunal remains, architectural features and finds, mainly from the late fourth century BC, providing insights into Alexander's campaigns in Central Asia [1]. Prior to Alexander's arrival, several important settlements in the region were located close to the modern town of Bandykhan/Bandixon, near the southern edge of the Baysun Basin [1–3]. Among the sites in this region is Gazimulla-Tepa (Bandykhan III, figure 1*a*), a city-type settlement around a citadel dating to the pre-Achaemenian period (Yaz IIB period), from the ninth to the sixth centuries BC [4,5]. The radiocarbon date falls into the ‘Hallstatt Plateau’ and therefore does not allow a precise calibrated date. However, the archaeological context is the pre-Achaemenian period, locally named Yaz IIB [3,6], so that a date before the middle of the sixth century BC can be established. It was only under Cyrus II (the Great) that Central Asia was conquered and integrated into the Achaemenian Empire. This occurred around 546–540 BC [7], so that the site of Gazimulla-Tepe must be placed before the mid-sixth century BC.

People living in this region largely relied on domesticated animal species as most faunal remains of that time in the region belong to species such as sheep, cattle and horses. Hunting still played a non-negligible role in their lives as a number of bones from wild mammals such as deers, gazelles and wild boars are found at the sites [8].

Ancient DNA approaches are a powerful addition to the zooarchaeological toolkit allowing to assign species, ancestry, biological sex and/or certain traits to faunal remains (e.g. [9,10]). However, most studies have focused on domesticated animals instead of wild species that were hunted. In this study, we sequenced DNA from an approximately 2700-year-old bovid bone fragment excavated at Gazimulla-Tepa during the year 2006 (figure 1*a*). The fragment (shown in an inset in figure 1*a*) was found among mostly domestic animal remains but we identify it as belonging to a goitered gazelle (*Gazella subgutturosa*). The goitered gazelle was among the most commonly hunted animals in prehistoric central and western Asia [8,11–13], and yet this is the first ancient DNA generated from the species allowing us to genetically categorize its relationships with modern gazelle populations.

## 2. Material and methods

### 2.1. DNA extraction and sequencing

We sampled approximately 150 mg of bone fragment from the humerus bone specimen (Collection Accession ID Gazimullah Y:2006 F:3, originally from Gazimulla-Tepa, Surkhandarya Province, Uzbekistan, figure 1*a*) at a dedicated ancient DNA facility at the Department of Organismal Biology of Uppsala University. Prior to sampling, the bone was mechanically cleaned with a Dremel drill and a 3.0% (w/v) sodium hypochlorite solution, followed by UVed water. We extracted DNA following [14].

After DNA extraction, we prepared two double-stranded Illumina DNA sequencing libraries following [15]. Between each step, the libraries were cleaned using Sera-Mag SPRI SpeedBeads (ThermoScientific) in 18% PEG-8000. Both libraries were sequenced independently at the National Genomics Infrastructure (NGI), SciLifeLab, Uppsala, on a NovaSeq 6000 Illumina DNA sequencer. Four additional faunal remains from Gazimulla-Tepa were screened for DNA; two of them yielded DNA and were identified as sheep.

### 2.2. Radiocarbon dating

**Figure 2.**
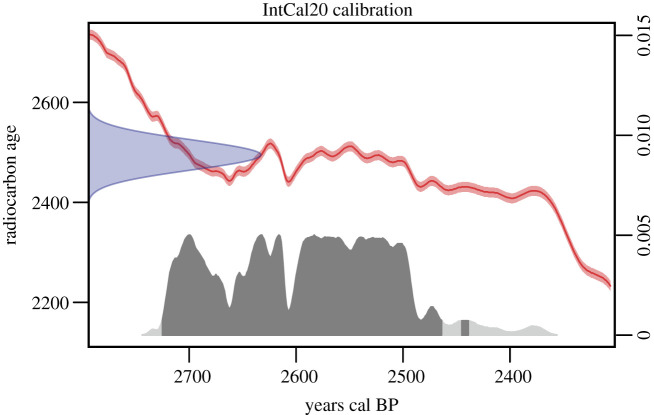
Calibrated radiocarbon age of AGAZ005 using *rcarbon* [16] and atmospheric data from IntCal20 [17].

An additional 980 mg of bone fragment was obtained from the initial bone sample for radiocarbon dating. The direct radiocarbon dating was performed at the Tandem Laboratory, Uppsala University, and the obtained radiocarbon age was 2495 ± 30. The date was then calibrated by using the R package *rcarbon* [16] with atmospheric data from [17] (figure 2).

### 2.3. Data processing and authentication

We processed the resulting sequencing data by removing sequencing adapters with CutAdapt [18], and merging the paired-end reads using Flash [19], requiring a minimum overlap of 11 bp and a maximum overlap of 150 bp. Due to the lack of a reference genome for the *Gazelle* genus, we mapped the merged reads to the sheep genome Oar_v3.1 (GenBank assembly accession: GCA_000298735.1) in order to assess nucleotide misincorporation and damage patterns. We mapped the merged reads to the sheep reference genome using BWA aln parameters established for ancient DNA studies (−l 1024 −n 0.01 −o 2) [20] and created sorted BAM files with samtools 1.12 [21]. We removed PCR duplicates with Picard's MarkDuplicate [22]. We also checked the biological sex of our sample using the Rx method [23] modified for a reference genome with 26 autosomes.

### 2.4. Species identification and mitochondrial genome assembly

We first used MEGAN v. c6.20.19 [24] to taxonomically assign the sequenced reads. Furthermore, we assembled the mitochondrial genome by mapping the reads to different mitochondrial reference genomes (table 1) using MIA (https://github.com/mpieva/mapping-iterative-assembler). MIA is an iterative assembler designed to be used with ancient DNA, improving alignment and consensus on chemically damaged DNA. The final consensus sequence for each assembly was called with each base having a minimum of threefold depth coverage and two-thirds base agreement.

**Table 1.** Sequencing information for both libraries. Mapped reads to the nuclear sheep genome, since there is no gazelle genome currently available.

sample	read pairs	merged reads	mapped reads	% mapped (%)	% duplicates (%)
AGAZ005 (lib1)	34 991 835	34 327 734	1 896 442	5.52	58
AGAZ005 (lib2)	21 264 728	21 204 583	514 280	2.59	43

### 2.5. Phylogenetic analysis

In order to investigate this newly assembled mitogenome, we downloaded an additional 66 complete mitochondrial genomes from NCBI from all major Bovidae clades (electronic supplementary material, table S1). We aligned all 67 mitogenomes using MAFFT [25] and inspected the alignment in SeaView 5.0.4 [26]. We reconstructed its phylogenetic tree through a Bayesian framework in MrBayes 3.2.7 [27] and also by using a maximum-likelihood approach in IQ-Tree [28,29]. We used a GTR + G nucleotide substitution model in both analyses [30,31]. In MrBayes, we ran three independent chains for two million generations ensuring a split frequency standard deviation below 0.01. In IQ-Tree, we ran 1000 bootstrap replicates (electronic supplementary material, figure S2).

## 3. Results

We sequenced about 56 million read pairs from two libraries. Approximately 4.3% of the merged reads mapped to the sheep reference genome (table 1). The DNA sequences show fragmentation and deamination patterns characteristic for ancient DNA damages [32] indicating authenticity of the sequence data (figure 1*b*,*c*). The sample was directly radiocarbon dated to 2725–2439 calBP (95.4% HDPI, figure 2) confirming its Iron Age origin.

### 3.1. Species identification

Initially, we attempted to identify the species of AGAZ005 by using the taxonomic assignment of sequences implemented in MEGAN [24]. Due to the lack of available Antilopinae sequencing data, BLAST-based taxon assignment indicates only the presence of Bovidae sequences, but does not allow for precise species identification (electronic supplementary material, figure S1). This analysis indicated the bone to be from a Bovidae specimen, and since this bone was found among several sheep bones [8], we mapped the merged reads to the sheep (*Ovis aries*) mitochondrial genome reference sequence NC_001941.1 using MIA (https://github.com/mpieva/mapping-iterative-assembler). Upon initial inspection, we found large stretches of poorly assembled regions of the mitochondrion, suggesting that the sequences do not originate from a sheep. We then performed a BLAST search (blastn) [33] using the partially assembled mitochondrial sequences. This allowed us to identify the fragmented assembly as goitered gazelle (*Gazella subgutturosa*, 97% identity, *E*-value = 1.9×10^−17^). We identified this individual as a female, according to the results from the Rx method adapted from [23] (Rx = 0.818, 95% CI: 0.8027–0.8345), which is based on the number of sequencing reads mapping to the sheep X chromosome.

### 3.2. Mitochondrial genome assembly

In order to assemble an improved mitochondrial genome for this specimen, we adopted a strategy that would also further strengthen our taxonomic identification results. We mapped the merged reads to multiple Bovidae species using MIA, as described above. We observed the number of mapping reads, and also how many MIA iterations were necessary for final convergence for the assembly (table 2). The final assembly using the *G*. *subgutturosa* reference sequence not only had the highest number of mapping reads, but also required the least amount of iterative mapping to converge.

**Table 2.** Mitochondrial genome assemblies statistics.

species	common name	mapped reads	average coverage	mapping iterations	accession number
*Gazella subgutturosa*	goitered gazelle	4500	23.212	4	JN632644.1
*Eudorcas rufifrons*	red-fronted gazelle	4171	21.896	30	MG603682.1
*Ovis aries*	sheep	3726	19.891	30	MW364895.1
*Rupicapra rupicapra*	chamois	3681	19.798	30	FJ207539
*Cephalophus dorsalis*	bay duiker	3589	19.356	30	JN632615.1
*Pseudois nayaur*	bharal	3553	18.830	30	JX101653.1
*Damaliscus pygargus*	bontebok	3487	18.976	30	FJ207530.1
*Oryx dammah*	scimitar oryx	3458	18.337	30	JN869311.1
*Capra ibex*	alpine ibex	n/a	n/a	did not converge	FJ207526.1
*Nanger dama*	dama gazelle	n/a	n/a	did not converge	JN632665.1

### 3.3. Phylogenetic placement

**Figure 3.**
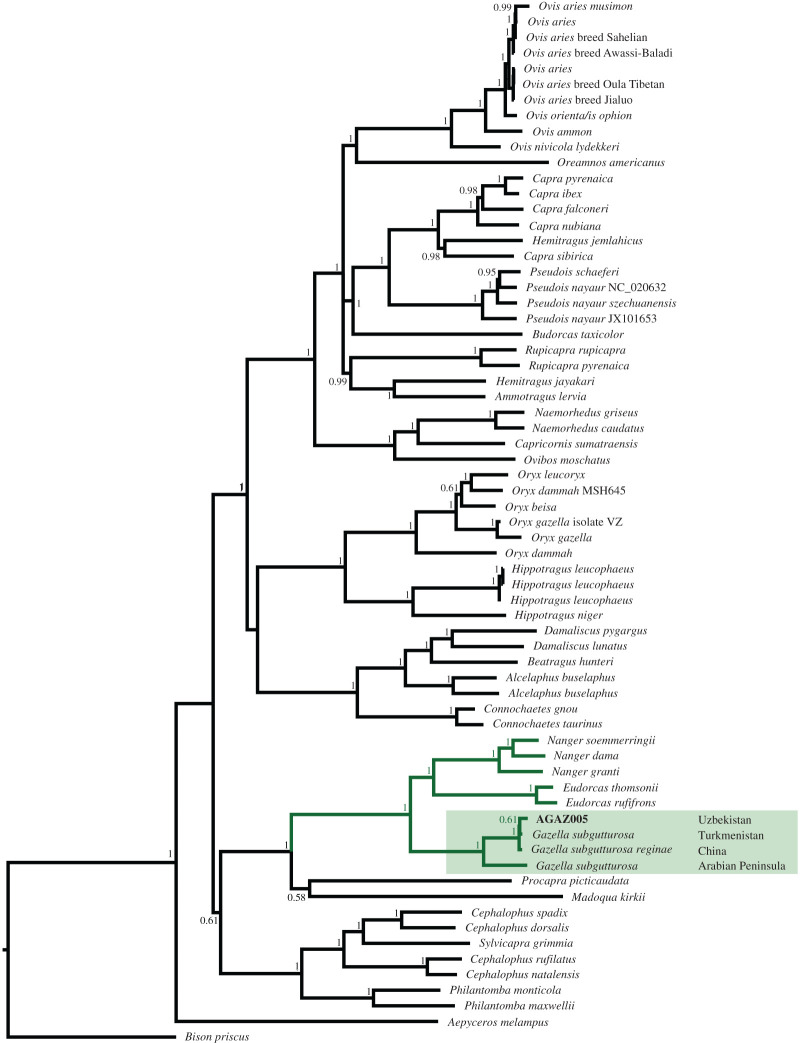
Bayesian inference phylogenetic tree for 68 mitochondrial genomes for a broad range of Bovidae species. The clade marked in green indicates all gazelle species represented in the tree, with our sample in bold. We added the general location for each *G*. *subgutturosa* specimen.

We built a phylogenetic tree under a Bayesian framework to investigate the relationship between our ancient sample and a wide range of Bovidae species (figure 3). On a general level, the present tree is unable to resolve the position of Cephalophini in relation to the gazelles. While the placement of Raphicerina and Procaprina in relation to Antilopina agrees with [34], support values are relatively low and disagree with the results from the maximum-likelihood tree (electronic supplementary material, figure S2). These patterns do not affect the species identification for AGAZ005, which is consistently clustered with modern gazelle sequences under both the Bayesian framework and the maximum-likelihood tree. This result further solidifies the finding that this bone fragment belongs to a *Gazella subgutturosa* individual. The phylogenetic tree places AGAZ005 within the *Gazella subgutturosa* clade, together with the other two *G. subgutturosa* individuals available for comparison from China and Turkmenistan [35,36]. Currently, the subspecies *G*. *subgutturosa yarkandensis* and *G*. *subgutturosa subgutturosa* exist in sympatry in Uzbekistan [37]. Unfortunately, we are currently unable to confidently identify which subspecies our specimen belongs to as the closest mitogenome from Turkmenistan does not have any subspecies information and additional genomes are not available for in-depth comparative analyses. Previous studies were based on partial mitochondrial sequences, and not complete mitochondria sequences, which limits the scope of analyses we could perform. Additional genomic data produced for the various *G*. *subgutturosa* subspecies will allow to disentangle the evolutionary history of this species. Especially, autosomal data has the potential to uncover population structure and gene flow between the subspecies, and our autosomal sequences could provide a temporal dimension for such studies, e.g. to investigate the early presence of *G*. *subgutturosa subgutturosa* with a later arrival of *G*. *subgutturosa yarkandensis* and potential gene flow as suggested by [37]. The *Gazella subgutturosa* subspecies nonetheless form a monophyletic clade within the Gazelle clade (marked in green in figure 3).

## 4. Discussion

Our study represents a direct genetic confirmation of a goitered gazelle excavated at a human settlement dating to Iron age Uzbekistan around 2725–2439 calBP (95.4% HDPI). This is consistent with morphological analyses of the faunal remains found in the region which lists goitered gazelles as the most common wild species at the neighbouring site Kurganzol dating mainly to the late fourth century BC [8]. Notably, most of the gazelle remains were highly fragmented, making morphological species identification challenging and highlighting the utility of archaeogenetics and other bioarchaeological approaches. Furthermore, a recent study using zooarchaeology by mass spectrometry (ZooMS, [38]) on faunal remains from Neolithic and Early Bronze Age Kyrgyzstan also classified some specimens found at human settlements as ‘deer/*Saiga*/gazelle’ [39].

Finding remains of wild animals in human settlements suggests that they were hunted, even though AGAZ005 itself does not display clear evidence for butchering or cooking. Hunting of gazelles at Iron Age Gazimulla-Tepa would be consistent with previous research showing that goitered gazelles have been subject to human hunting for several millennia across its entire range on the Asian continent with early evidence including the presumed mass-killings at ‘desert kites’ in the eastern Levant starting at least 11 000 BP [40,42]. Geographically closer to Gazimulla-Tepa, indications of gazelle remains were found at Obishir V, Kyrgyzstan, predating 4800 calBP [39], while numerous gazelle bone fragments with traces of butchering were found at Kurganzol about 50 km from Gazimulla-Tepa and postdating it by a few centuries [8,13,41].

As a result of habitat loss in combination with hunting and poaching, the species has been declining and today it is classified as ‘vulnerable’ by the IUCN. Our study takes a first step towards genomic and temporal studies of this wide-ranging ungulate. The lack of modern genome-wide data for goitered gazelles restricted the types of analysis we were able to perform. Our results indicate that this ancestral population is closely related to modern individuals that inhabit Turkmenistan and the Qaidam basin in China, but are unable to determine which subspecies of *G. subgutturosa* it belongs to. The structure and potential gene flow between subspecies as well as the presumed recent population decline, however, would make the goitered gazelle an interesting subject for broad geographic sampling and population genomic studies. Smaller scale studies based on microsatellite markers have already suggested local population structure [42]. Temporal genomic data could substantially enhance such studies of long-term processes. The genomic sequences generated for this project can be directly assigned to a point in time and space, and should therefore facilitate future studies of the population history of *G. subgutturosa*.

## Data Availability

Raw sequencing reads are available from the European Nucleotide Archive under accession PRJEB46820. The mitogenome for AGAZ005 (*Gazella subgutturosa*) was deposited at GenBank, accession no. ON468506. The data are provided in the electronic supplementary material [43].
